# Increase in Tumor Control and Normal Tissue Complication Probabilities in Advanced Head-and-Neck Cancer for Dose-Escalated Intensity-Modulated Photon and Proton Therapy

**DOI:** 10.3389/fonc.2015.00256

**Published:** 2015-11-20

**Authors:** Annika Jakobi, Armin Lühr, Kristin Stützer, Anna Bandurska-Luque, Steffen Löck, Mechthild Krause, Michael Baumann, Rosalind Perrin, Christian Richter

**Affiliations:** ^1^OncoRay – National Center for Radiation Research in Oncology, Faculty of Medicine, University Hospital Carl Gustav Carus, Technische Universität Dresden, Helmholtz-Zentrum Dresden – Rossendorf, Dresden, Germany; ^2^German Cancer Consortium (DKTK), Partner Site Dresden, Dresden, Germany; ^3^German Cancer Research Center (DKFZ), Heidelberg, Germany; ^4^Department of Radiation Oncology, Faculty of Medicine, University Hospital Carl Gustav Carus, Technische Universität Dresden, Dresden, Germany; ^5^Institute of Radiooncology, Helmholtz-Zentrum Dresden – Rossendorf, Dresden, Germany

**Keywords:** photon radiotherapy, proton radiotherapy, tumor control probability, normal tissue complication probability, head-and-neck cancer

## Abstract

**Introduction:**

Presently used radiochemotherapy regimens result in moderate local control rates for patients with advanced head-and-neck squamous cell carcinoma (HNSCC). Dose escalation (DE) may be an option to improve patient outcome, but may also increase the risk of toxicities in healthy tissue. The presented treatment planning study evaluated the feasibility of two DE levels for advanced HNSCC patients, planned with either intensity-modulated photon therapy (IMXT) or proton therapy (IMPT).

**Materials and methods:**

For 45 HNSCC patients, IMXT and IMPT treatment plans were created including DE via a simultaneous integrated boost (SIB) in the high-risk volume, while maintaining standard fractionation with 2 Gy per fraction in the remaining target volume. Two DE levels for the SIB were compared: 2.3 and 2.6 Gy. Treatment plan evaluation included assessment of tumor control probabilities (TCP) and normal tissue complication probabilities (NTCP).

**Results:**

An increase of approximately 10% in TCP was estimated between the DE levels. A pronounced high-dose rim surrounding the SIB volume was identified in IMXT treatment. Compared to IMPT, this extra dose slightly increased the TCP values and to a larger extent the NTCP values. For both modalities, the higher DE level led only to a small increase in NTCP values (mean differences <2%) in all models, except for the risk of aspiration, which increased on average by 8 and 6% with IMXT and IMPT, respectively, but showed a considerable patient dependence.

**Conclusion:**

Both DE levels appear applicable to patients with IMXT and IMPT since all calculated NTCP values, except for one, increased only little for the higher DE level. The estimated TCP increase is of relevant magnitude. The higher DE schedule needs to be investigated carefully in the setting of a prospective clinical trial, especially regarding toxicities caused by high local doses that lack a sound dose–response description, e.g., ulcers.

## Introduction

Standard of care for inoperable advanced head-and-neck squamous cell carcinoma (HNSCC) patients is concurrent radiochemotherapy, which today is still associated with a substantial recurrence rate (1, 2). Thus, an improvement of treatment outcome is desirable. Radiotherapy intensification to the primary tumor volume may improve patient outcome, since most recurring HNSCC after radiotherapy develop at the site of the initial primary tumor volume (3–5). Treatment intensification with radiation dose escalation (DE) is possible by applying non-uniform dose distributions. The simultaneous integrated boost (SIB) technique exploits the advantage of maintaining the treatment time – a critical factor in HNSCC radiotherapy (6–10). Radioresistant tumors may increasingly be identified by molecular profiling (11, 12), and radioresistant sub-regions within individual tumor volumes may be identified with functional imaging such as positron emission tomography (PET) (13, 14). Several groups have proposed dose painting of sub-volumes using hypoxia imaging with fluoromisonidazole (FMISO) PET (15–18). Since the capability of the dose painting approach to increase local tumor control is controversial, another approach is DE on the whole tumor volume (19, 20). However, treatment intensification may lead to an increase in side effects. Accordingly, higher dose conformity with proton therapy (PT) compared to advanced photon therapy (XT) may be beneficial. To estimate the overall benefit of treatment intensification, the probable gain in tumor control needs to be balanced against a potential increase in toxicity risk. This can be done in the treatment planning stage by comparing the resulting differences in tumor control probability (TCP) with those in normal tissue complication probability (NTCP).

Inhomogeneous dose prescriptions [e.g., different doses to gross tumor volume (GTV) and elective tumor volume] are driven by the clinical experience of a spatially heterogeneous dose–response in the target volume. Therefore, realistic modeling of TCP has to allow for dosimetric as well as radiobiological heterogeneity within the target. A recently presented TCP approach (21) provides dose–response relations for each of the considered target sub-volumes that base on clinical outcome data on the recurrence distribution in the tumor volume [e.g., Ref. (22)]. In contrast, if a homogeneous dose–response in the entire target volume was assumed, TCP estimates would suggest a low probability of treatment failures in the high-risk tumor sub-volume and most failures in the low-dose elective sub-volume (21), contradicting clinically observed data on failure patterns.

Regarding NTCP, in a previous work, we identified locally advanced HNSCC patients with substantial benefit from PT by comparing intensity-modulated XT (IMXT) with intensity-modulated PT (IMPT), focusing on patient sub-groups with similar primary tumor location (23). Therein, IMPT compared to IMXT showed the general capability to reduce NTCP. Moreover, we estimated the benefit of a mixed modality treatment (IMXT followed by IMPT for sequential boost treatment) by considering the NTCP reduction compared to IMXT alone revealing a minor effect in most of the patient cases (24). Following these studies, a prospective multi-centric clinical study is currently planned in our institution aiming at the evaluation of the effect of a 2.3 Gy DE in the treatment of advanced HNSCC.

In the present *in silico* study, we assessed the feasibility of a fractionation schedule for further treatment intensification via the SIB technique with a DE level of 2.6 Gy in comparison to the 2.3 Gy DE level applied in the previous work. We estimated TCP for these two DE levels and set the expected gain in relation to a potential increase in NTCP values.

## Materials and Methods

### Patient Data, Treatment Schedule, Volume Definition, Treatment Planning

Computed tomography (CT) and fluorodeoxyglucose (FDG) PET datasets of 45 patients treated between 2006 and 2013 at the University Hospital Dresden, Germany were available for the present analysis. Datasets consisted of a pre-treatment FDG PET/CT and a sequential FDG PET/CT recorded after approximately 20 fractions. All patients gave written consent for the use of their data. The study was approved by the institutional Ethics committee. A treatment schedule was defined that consists of two main treatment series planned on two different CT datasets: series I, a treatment series of 25 fractions for the elective clinical target volume (CTV_elec_), was planned on a baseline CT with 2 Gy per fraction plus a SIB starting at the eleventh fraction allowing for a hypoxia PET stratification based on a scan during treatment. This SIB volume (GTV_SIB-I_) was either defined as the GTV or, in the case of N3 status, as GTV and the N3 lymph nodes. A CTV_gross-I_ was generated by isotropic extension of 5–10 mm of the GTV_SIB-I_ and corrected for air cavities and bones if not infiltrated. Series II, a sequential boost of 11 fractions, was planned on a sequential PET/CT dataset taken after 20 treatment fractions. A dose of 2 Gy per fraction was prescribed to the CTV consisting of a geometrical expansion of the GTV and suspect lymph nodes (CTV_gross-II_). Additionally, the sequential boost contained a SIB to FDG-avid volumes inside the GTV identified on the FDG–PET scan after 20 treatment fractions (GTV_SIB-II_). Planning target volumes (PTV) were created for the CTV expanding 5 mm in cranio-caudal direction and 4 mm in plane, retaining a 3 mm distance to the external patient contour. Prescribed dose levels were 50 Gy to the CTV_elec_, 72 Gy to the CTV_gross_ and 79.8 Gy (DE1) or 87.6 Gy (DE2) to the SIB volume, depending on the DE level. The equivalent dose in 2 Gy fractions (EQD2) in the SIB volume was 81.3 Gy and 91.0 Gy, respectively, assuming an α/β ratio of 10 Gy. A constant correction factor of 1.1 was used for the higher relative biological effectiveness (RBE) of protons compared to photons, such that all values given in Gy actually mean Gy(RBE) for IMPT. Delineated organs at risk (OAR) were spinal cord, brain stem, ipsi- and contralateral parotid gland, ipsi- and contralateral brachial plexus, mucosa, swallowing muscles, larynx, esophagus, mandible, ipsi- and contralateral temporomandibular joints, ipsi- and contralateral submandibular and sublingual glands.

Intensity-modulated photon therapy treatment plans were based on seven equidistant 6 MV photon beams. A field reduction to five beams was considered for Series II for one-sided sequential boost volumes. IMPT treatment plans were based on a three field beam arrangement with beam angles of −40°, 40°, and 180°, but changes of these angles were possible for one-sided sequential boost volumes in Series II. Optimization goals for target structures were to irradiate at least 95% of the target volumes (PTV_elec_, PTV_gross_, GTV_SIB_) above 95% of the prescribed dose (*V*_95_ > 95%). Furthermore, volumes above 107% (*V*_107_) of the prescribed dose should be minimized. Such high-dose volume was accepted in the PTV if these were required to ensure the *V*_95_ in the GTV_SIB_. OAR constraints with priority over target goals were defined for spinal cord (*D*_max_ < 45 Gy), brain stem (*D*_max_ < 54 Gy), and brachial plexus (*D*_max_ < 72 Gy). To ensure that these constraints are met despite possible positioning uncertainties, the optimization was performed for these OARs with an additional margin of 3 mm considering the same dose constraint (brain stem and brachial plexus) or a slightly increased dose constraint (*D*_max_ < 48 Gy for spinal cord). For other OARs, doses were to be minimized without compromising target coverage. A more detailed description of the patient characteristics, treatment schedule, target definition, and treatment planning is presented in Jakobi et al. (23).

### Physical Dose Evaluation, TCP and NTCP Modeling

**Figure 1:**
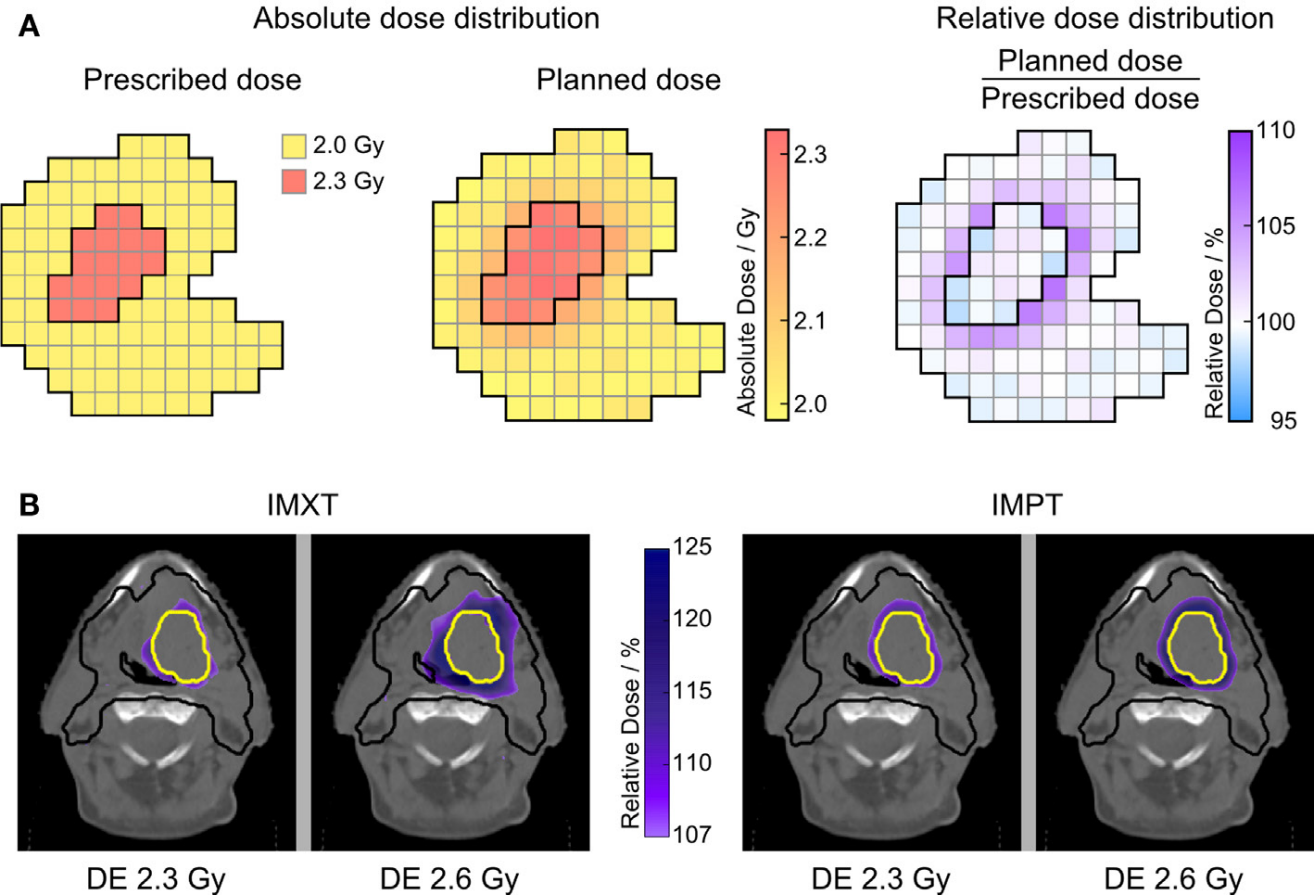
**Relative dose distributions**. **(A)** Illustration of calculating the voxel-wise relative dose distribution, exemplarily for DE level of 2.3 Gy. For the high DE level, the SIB volume is normalized to 2.6 Gy in the same way. **(B)** Relative dose distribution in the elective target only showing high-dose areas outside ICRU constraints (>107%). All four SIB treatment plans for one patient are shown for series I treatment, illustrating the larger high-dose rim around the SIB volume for the high DE level of 2.6 Gy. PTV_elec_ is outlined in black, GTV_SIB-I_ in yellow.

Dose parameters in the PTV and GTV_SIB_ were evaluated separately for both treatment series. For dose gradient evaluation, a relative dose distribution was created by normalizing each voxel to its prescribed target dose, as schematically shown in Figure 1A for the low DE level of 2.3 Gy. The dose was cumulated from both treatment series with a deformable image registration (DIR) on the pre-treatment CT and used to estimate TCP and NTCP. The DIR was validated in a previous study (25). In the target volume, the fractionation effect was considered by voxel-wise calculation of EQD2 (α/β = 10 Gy). For evaluation of NTCP, fractionation effect corrections were performed depending on model requirements.

**Figure 2:**
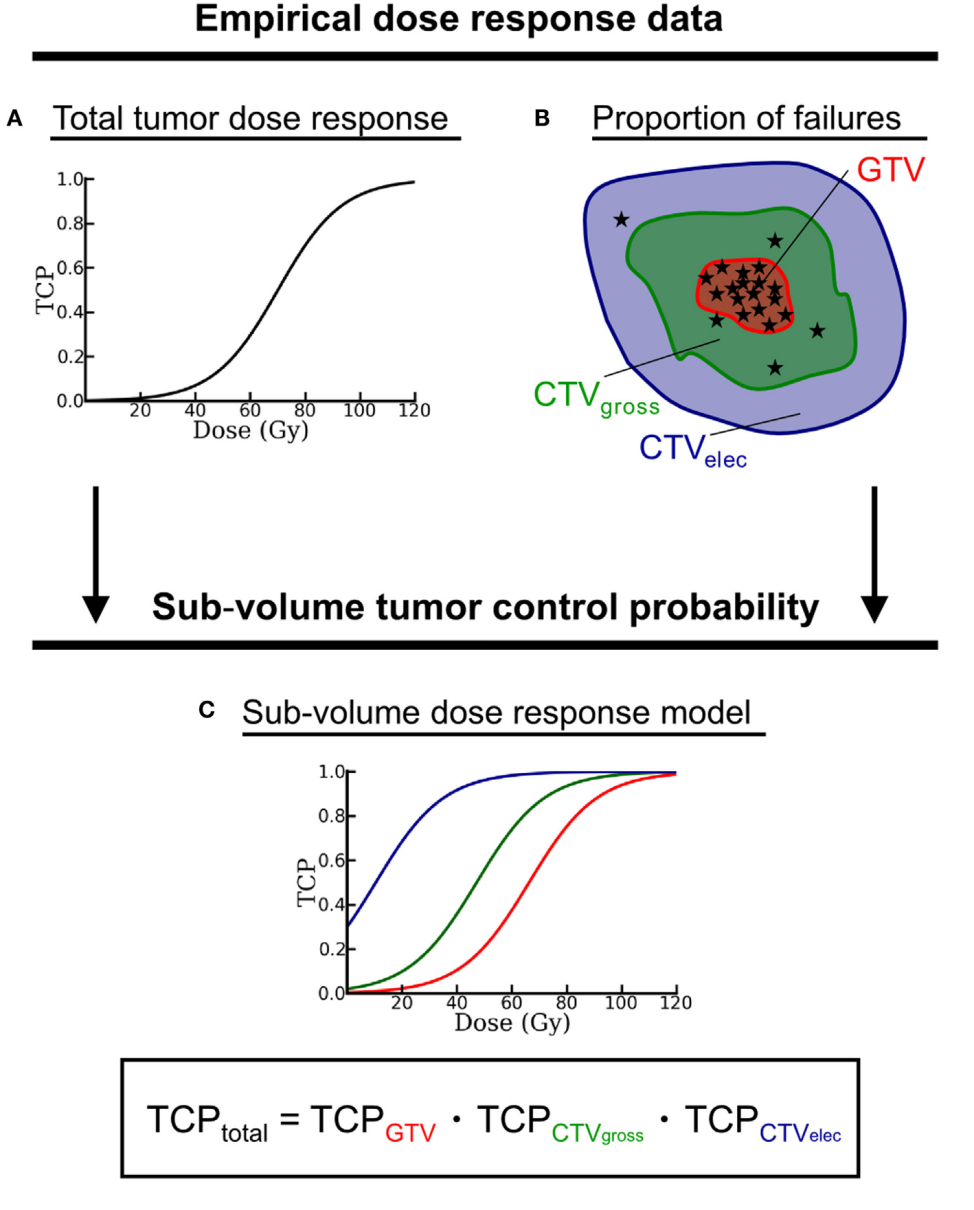
**Schematic drawing of the sub-volume TCP model approach**. Empirical dose–response data from comparable patient cohorts – given as **(A)** dose–response curve and **(B)** spatial distribution of local failures (represented by the asterisks) – serve as input to generate **(C)** one dose–response curve for each target sub-volume. The total TCP results from the product of all sub-volume TCP. Note, all target sub-volumes have to be disjoint. Therefore, inner sub-volumes are excluded from outer encompassing structures.

Tumor control probabilities modeling with local control as endpoint was based on an approach described by Lühr et al. [abstract in Ref. (21)]. This approach considers that the target volume consists of disjoint sub-volumes (schematically depicted in Figure 2), which differ in dose–response. The target structures CTV_elec_, CTV_gross_, and GTV_SIB_ were considered as target sub-volumes. To ensure that all sub-volumes were disjoint, inner sub-volumes were excluded from outer sub-volumes (e.g., GTV_SIB_ from CTV_gross_). According to clinically observed spatial failure patterns and the dose–response of a comparable patient cohort, the model approach assigns different dose–response curves to each sub-volume (cf. Figure 2) – each curve specified by its values for *D*_50_, the dose that yields a TCP of 50%, and γ_50_, the steepness of the TCP curve at the dose *D*_50_. In this study, the overall dose–response for homogeneous irradiation was approximated by *D*_50_ = 70 Gy and γ_50_ = 1.5 – assuming patients with advanced HNSCC – and by the relative proportions of local failures *f* = 0.80, 0.18, and 0.02 in GTV_SIB_, CTV_gross_, and CTV_elec_, respectively (22, 26). The *D*_50_ and γ_50_ parameters resulting from the sub-volume TCP approach (assuming the Poisson TCP model) for the three considered sub-volumes are listed in Table 1. TCP calculations were performed within the modeling framework of the recently developed ReCompare (REmote COMparison of PARticlE and photon plans) tool (27, 28).

**Table 1:** **TCP model parameters determining the dose–response in the target sub-volumes**.

Sub-volume	*D*_50_/Gy	γ_50_/%/%	*f*
Total	70.00^a^	1.50^a^	1.00
GTV_SIB_	66.79	1.43	0.80^a^
CTV_gross_	40.36	0.86	0.18^a^
CTV_elec_	6.73	0.14	0.02^a^

*They base on empirical total TCP parameters (*D*_50_, γ_50_) and on observed failure proportions *f* and were determined according to the sub-volume TCP approach*.

*^a^Values served as input data for the sub-volume model parameters *D*_50_ and γ_50_*.

Normal tissue complication probabilities values were calculated using recently published models for the following toxicities and endpoints: incidence of acute oral mucositis (grade ≥3); aspiration assessed by videofluoroscopy; xerostomia in terms of salivary flow reduction 12 months after therapy; subjective and objective swallowing dysfunctions; incidence risk of late larynx edema (grade ≥2); and trismus (jaw-opening <35 mm). Details on the model parameters can be found in Ref. (29–34) and as an overview in Jakobi et al. (23). Modeling the risk of a specific toxicity in a patient was skipped when a substantial portion of the NTCP-relevant organ was infiltrated by the tumor (physician’s decision).

To estimate the relative effect of the treatment intensification on tumor control and toxicity, individual patient matched-pair analyses of TCP and NTCP values were performed between the two different DE levels: ΔTCP = TCP_DE2_ − TCP_DE1_ and ΔNTCP = NTCP_DE2_ − NTCP_DE1_. The evaluation was carried out separately for IMXT and IMPT. Statistically significant differences between the DE levels were tested by two-sided paired *t*-tests with a significance level of 0.05. We analyzed the sensitivity of the TCP results by quantifying the dependence of ΔTCP on the input parameters *D*_50_ and γ_50_ for a homogeneous dose–response. One of the two input parameters was kept at its nominal value (70 Gy and 1.5, respectively) while the other parameter was varied within a certain range: 60 Gy ≤ *D*_50_ ≤ 80 Gy and 0.5 ≤ γ_50_ ≤ 3.0.

## Results

### Target Dose Evaluation

Dose coverage of the respective PTV (PTV_elec_, PTV_sequential boost_) and GTV_SIB_ structures (GTV_SIB-I_, GTV_SIB-II_) evaluated with *V*_95_ was similar for both DE schedules, independent of the treatment modality. The pursued minimum criterion (*V*_95_ > 95%) was fulfilled in all cases (IMXT and IMPT) except for *V*_95_ of GTV_SIB-II_ in both DE levels for one patient with a tumor close to a prioritized organ (brachial plexus).

High-dose volumes, evaluated by *V*_107_ in a structure composed of the respective PTV excluding the respective GTV_SIB_, the latter expanded by 5 mm, showed a significant increase between the two DE levels. For IMXT treatment, the mean patient-wise difference (±SD) *V*_107,DE2_ − *V*_107,DE1_ = 4.4 (±3.9)% (*p* < 0.001) is much larger than for IMPT with *V*_107,DE2_ − *V*_107,DE1_ = 0.1 (±1.6)% (*p* = 0.004). This is illustrated in Figure 1B by the relative dose distribution showing a large increase in dose above 107% surrounding the GTV_SIB-I_ for IMXT, while the increase for IMPT is small. *V*_107_ of the GTV_SIB_ was 0% in 44 of 45 patients.

### Evaluation of TCP

**Figure 3:**
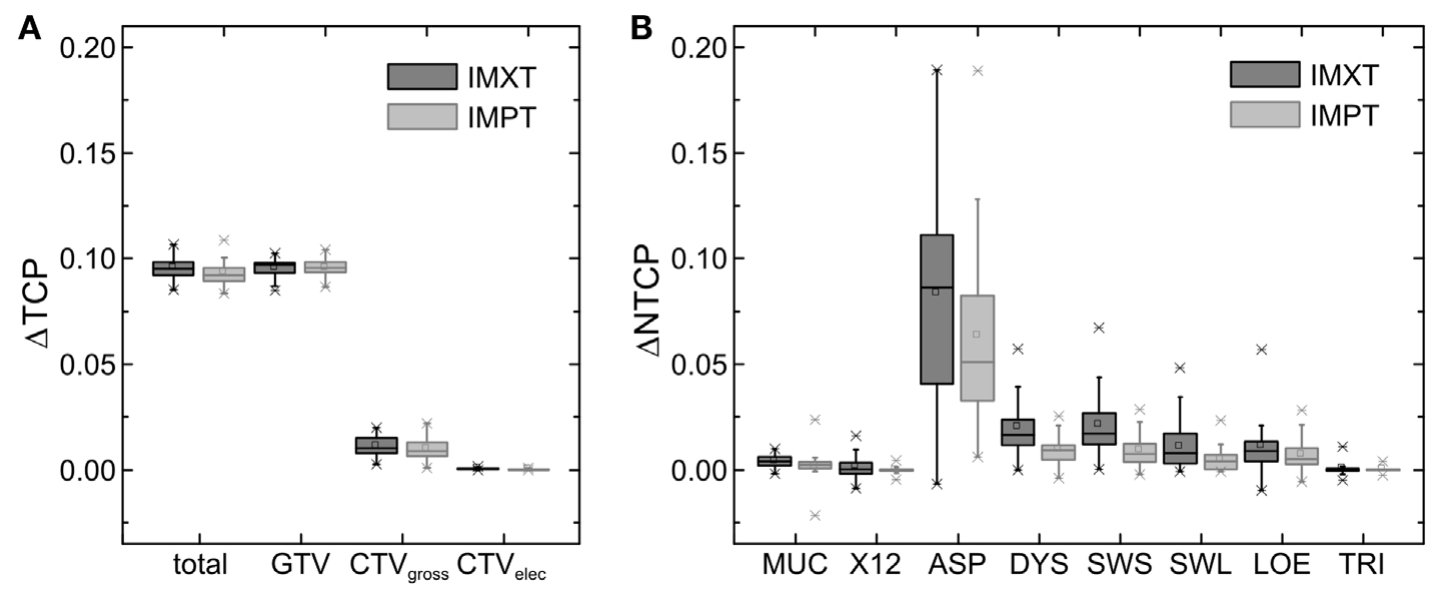
**Estimated differences between the two dose escalation levels for IMXT and IMPT: (A) ΔTCP and (B) ΔNTCP**. MUC, oral mucositis; X12, xerostomia after 12 months; ASP, aspiration; DYS, physician-rated swallowing dysfunction; SWS, patient-rated problems with swallowing solids; SWL, patient-rated problems with swallowing liquids; LOE, laryngeal edema; TRI, trismus.

Mean TCP values for all 45 HNSCC patients are given in the upper part of Table 2 for both DE levels. Significant differences between the two DE levels exist for all evaluated target structures for both modalities (*p* < 0.001). TCP values decreased from CTV_elec_ to CTV_gross-I_ to GTV_SIB-I_, i.e., from the outer to the inner target sub-volumes. The higher DE level led to a relevant increase in TCP values for the GTV_SIB-I_ (9.6% for both modalities) and the total TCP (9.6% with IMXT, 9.3% with IMPT), while the differences for CTV_gross-I_ and CTV_elec_ were small with mean differences of 1 and 0%, respectively, independent of the treatment modality (Figure 3A). This was expected since the DE with the SIB technique focused on the GTV, while the dose to CTV_elec_ and CTV_gross-I_ was targeted to remain stable between both DE levels. The small differences in TCP values for the CTV_gross-I_ between the DE levels resulted from the increase in dose that spilled out of the GTV into the surrounding CTV_gross-I_. TCP values for IMXT were in general slightly larger than for IMPT. This resulted from increased dose in the target regions around the GTV_SIB-I_ for IMXT, caused by its less conformal dose distribution of the integrated boost.

**Table 2:** **Mean (±1 SD) total and tumor sub-volume TCP values (upper rows) and NTCP values of the evaluated models (lower rows) for the two DE levels and both treatment modalities**.

	IMXT		IMPT	

	TCP_DE1_/%	TCP_DE2_/%	TCP_DE1_/%	TCP_DE2_/%
Total	66.3 ± 0.9	75.9 ± 1.3	65.5 ± 0.8	74.8 ± 1.3
GTV_SIB_	73.7 ± 0.8	83.2 ± 1.2	73.5 ± 0.9	83.1 ± 1.2
CTV_gross_	92.2 ± 0.6	93.3 ± 1.0	91.7 ± 0.5	92.7 ± 1.0
CTV_elec_	97.7 ± 0.4	97.7 ± 0.4	97.1 ± 0.5	97.1 ± 0.5

	**NTCP_DE1_/%**	**NTCP_DE2_/%**	**NTCP_DE1_/%**	**NTCP_DE2_/%**

Oral mucositis	45.8 ± 9.0	46.2 ± 9.2	40.0 ± 12.4	40.2 ± 12.5
Xerostomia	20.5 ± 10.9	20.6 ± 11.2	6.7 ± 4.6	6.7 ± 4.6
Aspiration	57.5 ± 23.2	65.8 ± 23.4	37.5 ± 25.1	43.8 ± 26.8
Dysphagia^a^	47.5 ± 11.4	49.5 ± 11.9	36.7 ± 13.1	37.7 ± 13.5
Swall. Solids^b^	34.4 ± 9.7	36.6 ± 10.5	25.7 ± 10.9	26.6 ± 11.4
Swall. Liquids^b^	10.3 ± 4.4	11.4 ± 5.2	9.0 ± 4.7	9.5 ± 5.1
Larynx edema	77.8 ± 25.0	79.0 ± 24.9	64.6 ± 36.9	65.4 ± 37.0
Trismus	30.9 ± 5.7	31.0 ± 5.8	26.0 ± 3.7	26.0 ± 3.8

*^a^Physician-rated swallowing dysfunction*.

*^b^Patient-rated swallowing problems of different severity*.

**Figure 4:**
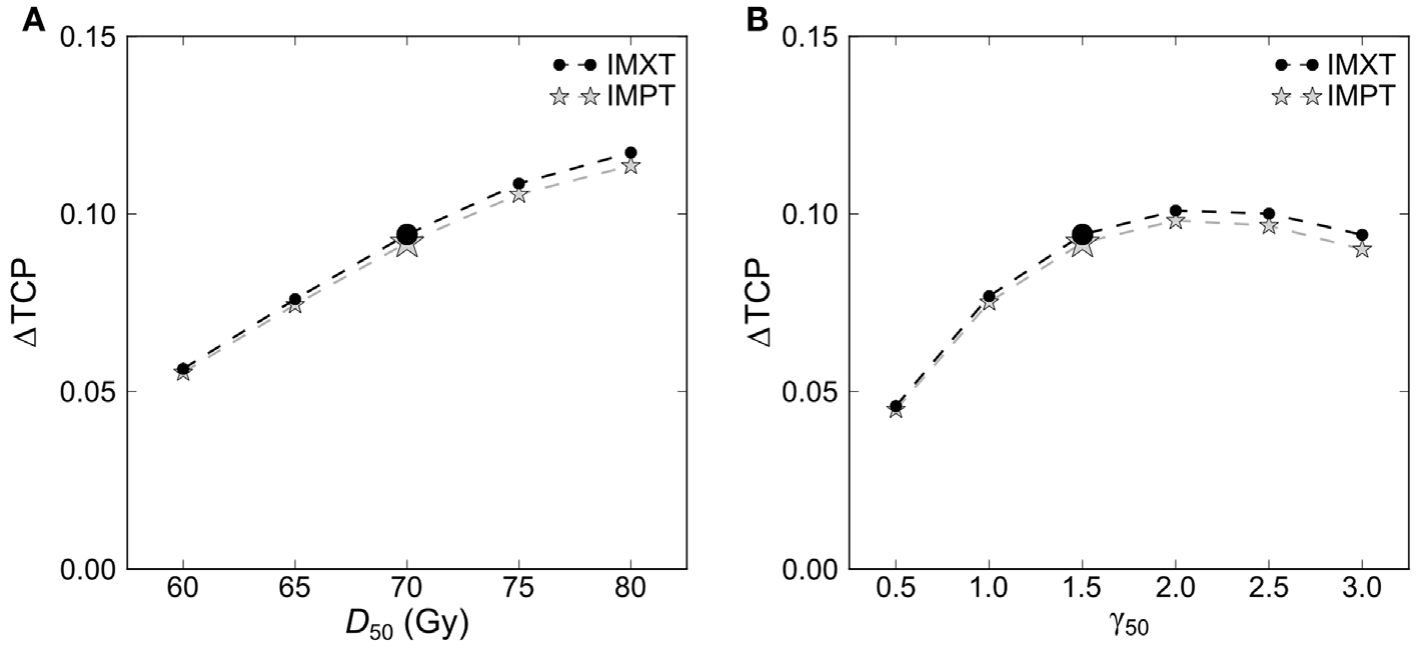
**Dependence of the difference in total tumor control probability ΔTCP between the two dose escalation levels on the model input parameters (A) *D*_50_ and (B) γ_50_**. Results are shown for IMXT and IMPT plans. The enlarged symbols mark the parameter values used in this study.

The estimated absolute TCP values depended on the employed model parameters. The mean increase in TCP from DE1 to DE2 was rather robust against the variation of the model parameters *D*_50_ and γ_50_ in intervals clinically reasonable for advanced HNSCC (Figure 4). For example, halving and doubling the slope parameter γ_50_ from a nominal value of 1.5–0.75 and 3 reduced the mean ΔTCP by about 0.03 and by 0.002, respectively. In comparison, the dependence of ΔTCP on *D*_50_ was stronger and the estimated gain in TCP between the DE levels increased monotonously for more radioresistant tumors (higher *D*_50_). The ΔTCP variation was very similar for IMXT and IMPT.

### Evaluation of NTCP

Mean NTCP values for all 45 HNSCC patients are given in the lower part of Table 2 for both DE levels. Absolute NTCP values were patient dependent and toxicity dependent. The integral dose in the patient external contour outside the target volume did not increase with the DE level. Similarly, dose to the OARs were in most cases only slightly increased. As a consequence, the influence of the DE level on NTCP values was almost negligible in most of the evaluated NTCP models with mean ΔNTCP values of approximately 1%. Only the risk of aspiration, modeled with the dose to the pharyngeal constrictor muscle (PCM), was substantially increased from DE1 to DE2 by on average (±SD) 8 (±4)% (*p* < 0.001) for all patients with IMXT and 6 (±4)% (*p* < 0.001) with IMPT (Figure 3B).

Additionally, the analysis revealed that all NTCP values for IMXT were larger than for IMPT (*p* < 0.001 for all evaluated models), especially for the risk of xerostomia and aspiration. This reflects the ability of IMPT to create more conformal dose distributions compared to IMXT. As a result, the ΔNTCP were also smaller in most cases for IMPT.

## Discussion

We conducted an analysis of the effect of DE for 45 HNSCC patients by comparing TCP and NTCP values of two different dose levels for IMXT as well as IMPT treatment in an *in silico* study. DE applied by the SIB technique allows for a confined treatment intensification focusing on the region of highest risk for recurrence with both, IMXT and IMPT. This is reflected by the estimated TCP values, which clearly increased for the GTV (the targeted region for treatment intensification) while the values remained almost unchanged for the surrounding target volumes, CTV_gross_ and CTV_elec_. Similarly, the dose to surrounding healthy tissues was only marginally increased for the higher DE level and the difference in NTCP values was practically negligible for all considered toxicities except for aspiration. The increase of estimated NTCP for aspiration resulted primarily from higher maximum doses in the PCM, since the model for aspiration uses a generalized equivalent uniform dose (gEUD) as input that is close to the maximum dose. Thus, an increase of high doses even in a small localized region of the PCM has a large impact on the NTCP value estimated by the employed aspiration model. Accordingly, setting a specific dose constraint for this organ in the treatment plan optimization may be appropriate in a DE study. All other employed NTCP models are based on mean organ dose as gEUD (or close to that) and their NTCP values were less sensitive to changes in local dose, leading to the observed small ΔNTCP values.

Treatment plans for PT possessed steeper dose gradients leading to a reduced high-dose spill into the tissue surrounding the SIB volume (high-dose rim) compared to IMXT plans. As a result, the ΔNTCP values were larger for IMXT than for IMPT. Also ΔTCP was slightly enhanced with IMXT for the two CTV sub-volumes. This difference in dose conformity together with the already lower NTCP level for IMPT let IMPT appear as a potential option for DE treatment in selected cases where IMXT leads to an unacceptable increase of NTCP values. However, for the lower DE level, DE1, with 2.3 Gy per fraction in the SIB volume (i.e., close to 2 Gy), the spill-over dose that increased the *V*_107_ in the CTV was still comparable between IMXT and IMPT. Thus, for such a low DE level the spill-over dose similarly affects the increase in toxicity risk for both treatment modalities. The small extent of spill-over for DE1 can be explained by the allowed dose variation in the two target volumes from 95% up to 107% of the prescribed dose, which is considered acceptable according to constraints of the International Commission on Radiation Units and Measurements (ICRU) (35). For example, a dose level of 2.3 Gy in the SIB volume requires (*V*_95_) a minimum of 2.19 Gy, while 2 Gy in the surrounding CTV permits (*V*_107_) a maximum of 2.14 Gy. Thus for DE1, both allowed dose limits are close together.

Based on the evaluated increase in toxicity risk via NTCP, a DE with a SIB of 2.6 Gy seems as feasible as a 2.3 Gy SIB for both modalities. Only for one toxicity endpoint (aspiration), an increase in risk was predicted by the NTCP models. At the same time, the expected benefit of the higher DE was a gain of about 10% in TCP which may be even higher for more radioresistant tumors (higher *D*_50_). The NTCP increase in aspiration of about the same magnitude is of clinical concern, as aspiration pneumonia may be the consequence and thus might be unacceptable in this relation, calling for a well-chosen dose limit for the PCM. The overall small increase in toxicity risk for most models for the evaluated DE level is in accordance with other published studies. Isotoxic DE from 70 Gy to comparable dose levels was rated feasible with a SIB in a small treatment planning study by Thorwarth et al. (36), where a DE of 50% (DE2 in the present study would corresponds to about 25%) was assessed as upper limit by evaluation of dosimetric data for a smaller number of OARs. Leclerc et al. (37) demonstrated the clinical applicability of a SIB with 2.5 Gy per fraction in a multi-centric phase I–II study, however, with a reduced total dose of 75 Gy (EQD2 = 78.1 Gy) and less advanced tumor stages.

This is the first study that employs the sub-volume TCP model to analyze the potential gain of different DE levels limited to a high-risk target sub-volume. The approach builds on established empirical knowledge on dose–response for homogeneous dose irradiation and corresponding spatially heterogeneous patterns of treatment failures. Recently, Vogelius et al. used a conceptually similar approach to analyze the potential of a data-driven dose-painting strategy for HNSCC (38). Assuming *D*_50_ to be close to 60.5 Gy, they found a substantial increase in local control with an estimated TCP of 89% for spatially optimized dose prescriptions. Considering the same *D*_50_ parameter, this TCP value is in good agreement with a TCP of about 87% estimated with the approach of the current study for the high DE level DE2. An ongoing Danish clinical trial that tests the data-driven dose-painting approach is supposed to provide clinical evidence that further supports the used TCP model.

Normal tissue complication probability evaluation was restricted to published toxicity models. The only toxicity, for which an increased risk of NTCP was found, differed from the others in modeling by being sensitive to local high-dose levels. This may also occur for other dose limiting toxicities, e.g., for ulceration of tissue, which was shown to be sensitive to high local doses within small volumes (39). Treatment planning studies evaluating the feasibility of DE in the view of potential side effects are limited to known dose–response effects and are a first step allowing for an ethically justifiable clinical trial. Thus, the theoretical feasibility of the DE schedule, demonstrated with the presented treatment planning study, needs to be carefully validated in a clinical setting.

A limitation of the presented analysis is the use of nominal dose distributions. As Müller et al. (40) and Góra et al. (41) showed, IMPT treatment plans are more prone to dose distortions originating from anatomical changes of the patients. Such changes can decrease the dose conformity to the target volume and thus deteriorate the TCP. Furthermore, they can result in increased dose to nearby healthy tissues, increasing the NTCP. The presented treatment planning study design included a one-step adaptation strategy to reduce the effects of patient anatomy changes on the dose distribution. Changes in anatomy would impose in a similar way on the dose distributions of the two compared DE levels, reducing their effect on the differences evaluated in the present study. However, the adaptation approach introduces an additional uncertainty by using a DIR for dose accumulation. Again, this uncertainty affects both DE levels in a similar way, reducing its influence on the difference values. In a clinical setting, close consideration is required to limit the effect of anatomical changes, e.g., by implementing plan adaptation protocols.

Another limitation originates from uncertainties connected to the modeling of the TCP and NTCP values. As a consequence, the results need to be interpreted carefully, especially, when absolute TCP and NTCP values are considered. However, this study focused on differences between model values for the two DE levels. Such relative results tend to be more robust, as some uncertainties may affect the absolute NTCP and TCP values in a similar way, having a minor effect on the differences. For example, the *D*_50_ parameter sensitivity analysis, which covered a broad *D*_50_ interval – i.e., a large range of patient characteristics – led to a variation of the absolute TCP on the order of 35% (e.g., DE1 of IMXT: from 83 to 47%). Evaluating for the same data the impact on ΔTCP resulted in a variation of only 7% (cf. Figure 4). For NTCP values a comprehensive parameter sensitivity analysis was beyond the scope of this study. A case study for the physician-rated dysphagia model showed that for IMXT a doubling or halving of the input parameters led to a relative change of mean ΔNTCP of approximately 50%, i.e., from 2 to 3% and to 1%, respectively. However, absolute NTCP values were similar to values found in other publications using these models, and thus the model parameters seem to be reliable for the presented patient cohort (31, 42). Consequently, even for less favorable model parameters (low *D*_50_ or γ_50_), a substantial ΔTCP increase of 5% between the DE levels was estimated, while ΔNTCP remained at a smaller level in the case study. Assuming for the other models an effect of similar magnitude, and considering the small NTCP values for most patients, model parameter changes would lead to only little changes regarding the presented statements.

## Conclusion

The presented *in silico* study evaluated two treatment intensification strategies differing in the SIB dose level to the high-risk tumor sub-volume for advanced HNSCC patients. The increase of the DE level from 2.3 to 2.6 Gy per fraction was feasible with IMXT and IMPT retaining integral dose and NTCP values of all but one endpoint. For aspiration, an increase in estimated toxicity risk was identified. The relevant increase in TCP between the DE levels originated from a higher TCP in the SIB volume, which is of the same order of magnitude as the estimated increase in aspiration toxicity and much higher than the increase of the other evaluated toxicities. Weighing the large TCP gain against the little NTCP increase of all evaluated models, the use of the higher DE level may be beneficial from a clinical point of view, except for those situations, where aspiration is of clinical concern. Since the analysis was restricted to available toxicity models, these findings need to be further investigated in prospective clinical studies.

## Author Contributions

AJ participated in the design of the treatment planning study, chose the NTCP models, performed the treatment planning, physical dose and NTCP analysis and interpretation of the data, and drafted the manuscript. AL created the TCP model, performed the TCP calculations, analyzed the TCP data, and drafted the manuscript. KS performed parts of the treatment planning, the processing (dose summation, DIR, NTCP calculations) and interpretation of the data for the physical dose, and NTCP analysis. AB-L participated in the design of the treatment planning study, performed the delineation of volumes for treatment planning, and advised in the NTCP model choice. SL processed the data for the TCP calculation (dose summation, TCP calculation) and calculated the relative dose distributions. MK, MB, RP, and CR participated in the design of the treatment planning study and provided general supervision. MK and CR contributed to the data interpretation. All authors revised and approved the manuscript.

## Conflict of Interest Statement

The authors declare that the research was conducted in the absence of any commercial or financial relationships that could be construed as a potential conflict of interest.
